# Aragon workers’ health study – design and cohort description

**DOI:** 10.1186/1471-2261-12-45

**Published:** 2012-06-19

**Authors:** José A Casasnovas, Victor Alcaide, Fernando Civeira, Eliseo Guallar, Borja Ibañez, Jesús Jiménez Borreguero, Martin Laclaustra, Montserrat León, José Luis Peñalvo, José M Ordovás, Miguel Pocovi, Ginés Sanz, Valentín Fuster

**Affiliations:** 1 Cardiovascular Research Unit, Instituto Aragonés de Ciencias de la Salud (I + CS), Zaragoza, Spain; 2 Prevention Depatment, General Motors Spain, Figueruelas, Spain; 3 Lipids Unit and Molecular Research Laboratory, Hospital Universitario Miguel Servet, Instituto Aragonés de Ciencias de Salud (I + CS), Zaragoza, Spain; 4 Department of Epidemiology, Atherothrombosis, and Imaging, National Center for Cardiovascular Research (CNIC), Madrid, Spain; 5 Welch Center for Prevention, Epidemiology, and Clinical Research, Johns Hopkins University Bloomberg School of Public Health, 2024 E. Monument St., Room 2-639, Baltimore, MD, 21205, USA; 6 Cardiovascular Institute, Hospital Clínico San Carlos, Madrid, Spain; 7 Department of Cardiology, Hospital de la Princesa, Madrid, Spain; 8 Nutrition and Genomics Laboratory, US Department of Agriculture Human Nutrition Research Center on Aging, Tufts University, Boston, USA; 9 Madrid Institute for Advanced Studies on Food (IMDEA Food), Madrid, Spain; 10 Department of Biochemistry, Molecular and Cell Biology, University of Zaragoza, Zaragoza, Spain; 11 Department of Biochemistry, Molecular and Cell Biology, University of Zaragoza, Instituto Aragonés de Ciencias de la Salud (I + CS), Zaragoza, Spain; 12 Centre for Biomedical Network Research on Rare Diseases (CIBERER), Zaragoza, Spain; 13 Zena and Michael A. Wiener Cardiovascular Institute, and the Marie-Josee and Henry R. Kravis Cardiovascular Health Center, The Mount Sinai School of Medicine, New York, USA

## Abstract

**Background:**

Spain, a Mediterranean country with relatively low rates of coronary heart disease, has a high prevalence of traditional cardiovascular risk factors and is experiencing a severe epidemic of overweight/obesity. We designed the Aragon Workers’ Health Study (AWHS) to characterize the factors associated with metabolic abnormalities and subclinical atherosclerosis in a middle aged population in Spain free of clinical cardiovascular disease. The objective of this paper is to describe the study design, aims and baseline characteristics of participants in the AWHS.

**Methods/Design:**

Longitudinal cohort study based on the annual health exams of 5,400 workers of a car assembly plant in Figueruelas (Zaragoza, Spain). Study participants were recruited during a standardized clinical exam in 2009–2010 (participation rate 95.6%). Study participants will undergo annual clinical exams and laboratory assays, and baseline and triennial collection of biological materials for biobanking and cardiovascular imaging exams (carotid, femoral and abdominal ultrasonography, coronary calcium score, and ankle-arm blood pressure index). Participants will be followed-up for 10 years.

**Results:**

The average (SD) age, body mass index, and waist circumference were 49.3 (8.7) years, 27.7 (3.6) kg/m^2^ and 97.2 (9.9) cm, respectively, among males (N = 5,048), and 40.8 (11.6) years, 24.4 (3.8) kg/m^2^, and 81.9 (9.9) cm, among females (N = 351). The prevalence of overweight, obesity, current smoking, hypertension, hypercholesterolemia, and diabetes were 55.0, 23.1, 37.1, 40.3, 75.0, and 7.4%, respectively, among males, and 23.7, 8.3, 45.0, 12.1, 59.5, and 0.6%, respectively, among females. In the initial 587 study participants who completed all imaging exams (94.5% male), the prevalence of carotid plaque, femoral plaque, coronary calcium score >1 to 100, and coronary calcium score >100 was 30.3, 56.9, 27.0, and 8.8%, respectively. 67.7% of study participants had at least one plaque in the carotid or femoral arteries.

**Discussion:**

Baseline data from the AWHS show a high prevalence of cardiovascular risk factors and of sublinical atherosclerosis. Follow-up of this cohort will allow the assessment of subclinical atherosclerosis progression and the link of disease progression to traditional and emergent risk factors.

## Background

Cardiovascular disease (CVD) is the first cause of death worldwide, but there are substantial differences in its incidence, prevalence and mortality across countries and ethnicities [1-3]. Spain, a Mediterranean country with low rates of coronary heart disease (CHD) compared to other Western countries [4-7], has a paradoxical high prevalence of traditional cardiovascular risk factors, including dyslipidemia, hypertension, smoking, and diabetes [5,6,8-11]. Furthermore, Spain is experiencing a severe epidemic of overweight/obesity [12,13] that will likely increase the rate of CHD and other obesity-related pathologies. The reasons for the discrepancy between the risk factor profile and the incidence of CHD in Spain are unclear and deserve additional study [14]. In addition, there is substantial interest in understanding how a population-wide increase in adiposity affects atherosclerosis and cardiovascular endpoints in a Western country with relatively low background levels of CHD.

Recent advances in imaging techniques provide a unique opportunity to understand the relation between risk factors and subclinical CVD at the population level. Calcium coronary scoring increases risk discrimination compared to risk equations based on traditional cardiovascular risk factors [15]. Moreover, carotid [16] and aortic ultrasound scans [17], and the ankle-brachial blood pressure index [18] provide added information on the presence of subclinical atherosclerosis in non-coronary vascular territories [19]. In this context, the specific genetic and environmental factors associated with the presence and the progression of subclinical atherosclerosis in Mediterranean populations with high prevalence of risk factors are still relatively unexplored, particularly in middle-aged populations that may have developed subclinical disease but not clinical symptoms.

In this paper, we describe the objectives, the methods used for data collection and participant follow-up, and the baseline characteristics of the Aragon Workers’ Health Study (AWHS), a longitudinal cohort study to characterize the factors associated with metabolic abnormalities and subclinical atherosclerosis in a middle aged population in Spain free of clinical CVD.

## Methods/Design

### Objectives of the Aragon workers’ health study

The AWHS was designed to evaluate the trajectories of traditional and emergent CVD risk factors and their association with the prevalence and progression of subclinical atherosclerosis in a population of middle-aged men and women in Spain. The study involves annual evaluation of cardiovascular risk factors and clinical endpoints together with baseline and triennial blood and urine sampling for biobanking and imaging of subclinical atherosclerosis in a cohort of over 5,000 workers of a large car assembly plant in Figueruelas (Zaragoza, Spain). The study started in February 2009 and completed enrollment in December 2010. Active follow-up of cohort participants will extend through 2020.

The specific aims of AWHS are to establish the research infrastructure required for a longitudinal cohort study, including setting up a biobank of repeated biological samples to conduct future assays in stored serum, plasma, whole blood, urine, and DNA; to identify new genetic, behavioral, and environmental determinants of the progression of adiposity and of the development of metabolic abnormalities and cardiovascular risk factors; to characterize the prevalence and progression of subclinical CVD through non-invasive imaging techniques and their genetic, behavioral, and environmental determinants; to interact with external investigators to promote the use of the study database and stored materials for ancillary studies; and to disseminate the study findings to the scientific community, to public health authorities, and to the general public.

### Sources of support

AWHS is funded through a collaborative agreement between the Instituto Aragonés de Ciencias de la Salud (I + CS) of the regional Government of Aragón, the National Center for Cardiovascular Research (CNIC) of the Instituto de Salud Carlos III, and General Motors Spain. The study was approved by the governing and scientific boards of I + CS and CNIC, and by the management and the workers’ representatives of General Motors Spain.

### Study design and population

**Figure 1:**
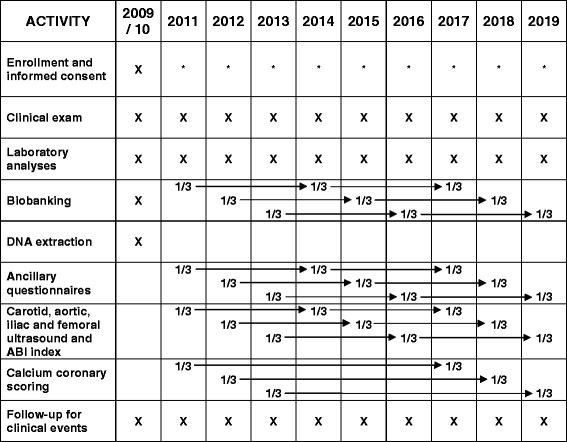
Schedule of data collection, Aragon Workers Health Study.

AWHS is a prospective, longitudinal cohort study based on the annual health exams of the workers of the General Motors Spain automobile assembly plant located in Figueruelas (Zaragoza, Spain). Each year, factory workers undergo a standardized clinical exam. Starting in February 2009, factory workers were asked for consent to participate in AWHS by allowing use of their annual health exam data, by completing additional questionnaires on cardiovascular and lifestyle risk factors, by providing blood and urine samples for the study biobank, and by participating in triennial exams to assess the presence of subclinical atherosclerosis. The schedule of data collection in AWHS is summarized in Figure 1. According to the study protocol, workers who did not attend the annual exam during the first year or who start working in the factory after the study started will be offered the possibility of entering the study in future exams. Workers are excluded from the cohort if they have clinically overt CVD, or a major clinical condition limiting survival to <3 years at baseline. The study was approved by the central Institutional Review Board of Aragón (CEICA). All study participants provided written informed consent.

### Data collection

Data collection is organized around the annual medical exam that the Medical Services of General Motors Spain provide to all workers. The clinical exam follows standardized protocols using validated procedures and instruments. Data collection at the annual medical exams is conducted by the physicians and nurses of the Medical Services of General Motors Spain, who underwent training and standardization programs organized by the study investigators. All study procedures are described in the standard operating procedures. Compliance with study procedures is routinely monitored and deviations are corrected. The study conforms to the ISO9001-2008 quality standard.

At each annual exam, study participants provide a clinical history, including clinical events and hospitalizations over the past year and current medication use, and undergo a physical exam, including anthropometry (height, weight, and waist circumference), blood pressure measurements and heart rate. Blood pressure is measured three consecutive times using an automatic oscillometric sphygmomanometer OMRON M10-IT (OMRON Healthcare Co. Ltd., Japan) with the participant sitting after a 5-min rest. Each participant also provided a sample of blood and urine after overnight (>8 h) fasting for laboratory analyses and for biobanking.

Each year, one random third of study participants 40 – 55 years of age at baseline will be selected for subclinical atherosclerosis imaging and for additional questionnaires of cardiovascular and lifestyle factors, including the Spanish validated versions of the Nurses’ Health Study and Health Professionals’ Follow-up semi-quantitative food frequency [20] and physical activity questionnaires [21], and the Center for Epidemiological Studies – Depression (CES-D) scale [22].

### Subclinical atherosclerosis imaging

Imaging of subclinical atherosclerosis is performed at the AWHS Clinic located at the Hospital Universitario Miguel Servet in Zaragoza, Spain. Calcium coronary scoring is performed using non-contrast ECG gated prospective acquisition by a 16 multidetector computed tomography scanner (Philips). We will evaluate the progression/regression of calcium score as a continuous variable. In addition, we will categorize coronary calcium scores in 3 mutually exclusive groups: none; Agatston score ≥1 to 100 and <75^th^ percentile of the score in the MESA population for the same age and sex [23]; and Agatston score >100 or ≥75^th^ percentile of the score in the MESA population for the same age and sex. We selected Agatston scores >100 or ≥75^th^ percentile of the age- and sex-specific distributions because these have been the lower lower levels of coronary calcium associated with mild increases in coronary heart disease event rates [23].

Carotid intima-media thickness and the presence of carotid plaque in both carotid arteries is determined using an ultrasound system IU22 Philips. Ultrasound images are acquired by linear high frequency 2-dimensional (2D) and 3-dimensional (3D) probes following the protocol of the Bioimage Study [19]. Carotid plaque is defined as a focal structure that protrudes into the lumen of the carotid artery at least 0.5 mm or ≥50% thicker than the surrounding intima-media [24]. Intima-media thickness is considered abnormal if it is ≥75^th^ percentile of the age- and sex of reference population [25].

Abdominal aorta aneurysms (AAA) are identified using abdominal ultrasound with a linear transducer [26] AAA are defined as localized dilatations of the abdominal aorta ≥50% greater than the aortic diameter or with a diameter ≥30 mm in cross-sectional view [24]. 2D and 3D ultrasound imaging of the right and left femoral and iliac arteries are also be performed by using linear array transducers (Philips) [27]. Mean common femoral intima-media thickness is calculated as the average of the distances between the intima and media layers by ultrasound in a 2-cm segment proximal to the bifurcation. Plaques are defined as in the carotid arteries. In all cases plaques are recorded in both longitudinal and transverse planes to take into consideration circumferential asymmetry.

The ankle-brachial index is calculated from resting blood pressure measurements in the brachial arteries in both arms and the posterior tibial and *dorsalis pedis* arteries in both legs. We will consider abnormal an ankle-brachial index ≤0.9 in at least one leg.

In AWHS, imaging techniques are applied to study participants who are 40–55 years old at baseline. Ultrasound measures of the carotid, aortic, femoral and iliac arteries and ankle-brachial blood pressure index are measured in a random third of eligible participants each year during years 2–4 of the study, and then repeated in years 5–7 and 8–10 of the study. Coronary calcium will be measured in years 2–4 of the study in a random third of eligible participants each year and then repeated in years 8–10 of the study. For each eligible participant, we will thus be able to study the progression/regression of subclinical atherosclerosis over 6 years of follow-up.

### Laboratory analysis

Each year, study participants provide a blood and urine samples after overnight fasting. A battery of laboratory tests is performed annually in all workers at the laboratory of the Medical Services of General Motors Spain. Fasting serum glucose, triglycerides, total cholesterol and HDL-cholesterol are measured by spectrophotometry (Chemical Analyzer ILAB 650, Instrumentation Laboratory), serum apolipoproteins AI and B by kinetic nephelometry (Immunochemistry Analyzer IMMAGE 800, Beckman Coulter), and fasting serum insulin by immunoenzymatic chemiluminiscence (Access Immunoassay System, Beckman Coulter). Whole blood HbA1c is measured by reverse-phase cationic exchange chromatography and quantification by double wave-length colorimetry quantification (Analyzer ADAMS A1c HA-810, Arkray Factory).

AWHS participates in two external quality control programs: the national quality control program of the Spanish Society for Clinical Chemistry (SEQC) for glucose, triglycerides, total cholesterol, HDL-cholesterol, apolipoprotein A1, and apolipoprotein B, and insulin, and an international quality control program by BIO-RAD for glucose, total cholesterol, and HDL-cholesterol.

Blood and urine samples are processed the same day of extraction. Biological samples are stored from all cohort participants at baseline and for the one-third subsample selected for imaging techniques each year. For biobanking, about 15 ml of EDTA blood and 5 ml of blood without anticoagulant for serum preparation are fractionated into plasma, serum and whole blood and stored in 1 ml dot-coded metal-free cryotubes at −80 °C. Spot (casual) urine samples are also collected and stored in 5 ml vials at −80 °C. Genomic DNA is extracted from peripheral blood cells by use of commercial reagents (FlexiGene DNA Kit, QIAGEN, USA). DNA purity and concentration is determined by absorbance at 260 nm (A260) and 280 nm (A280) using Nanovue (GE Healthcare, Munich Germany). DNA is stored in 0.3 ml vials at −80 °C. Biological specimens for each participant are stored in two geographically separated repositories.

### Risk factor definition

Definitions of cardiovascular risk factors were based on European Guidelines [28,29]. Hypertension was defined as a measured blood pressure ≥140/90 mmHg (130/80 in participants with diabetes) or current use of antihypertensive medication. Hypercholesterolemia was defined as a total cholesterol level ≥190 mg/dL (4.9 mmol/L) or current use of lipid lowering medication. Diabetes was defined as a diagnosis of diabetes in the clinical record, current use of antidiabetic medication, a fasting serum glucose ≥126 mg/dL (7.0 mmol/L), or an HbA1c ≥6.5% [30].

### Cohort follow-up

Follow-up of study participants is based primarily on the annual medical exams at the factory. In addition to these exams, the Medical Services of General Motors Spain routinely collect information on any clinical or health-related event occurring to the workers during the study period. Furthermore, an Absenteeism Center at General Motors Spain tracks medical leaves or disabilities of all workers. Finally, when workers who have been on medical leave for >30 days return to work, they have to undergo a mandatory clinical exam at the factory’s Medical Services to collect information on the possible causes and sequelae of the process responsible for the medical leave. All diagnosis collected through the Medical Services or the Absenteeism Center are revised and coded according to standardized criteria by the study Endpoints Committee.

For workers who discontinue employment during the study period, we will register the reason for discontinuation (e.g. retirement) and its possible relation to health events, and we will offer study participant the possibility of continuing the annual exams and the triennial evaluations of subclinical atherosclerosis at the AWHS Clinic located in the Hospital Miguel Servet in Zaragoza, Spain.

### Statistical methods

AWHS data are integrated in a relational database that also provides support to the study biobank and the study research and dissemination processes. Database management and related processes, including anonymization, security, back-ups, documentation, and data checks and corrections, are documented in detail. The descriptive analyses of baseline data presented in this paper are based in means and standard deviations for continuous variables and count and proportions for categorical variables. Statistical analyses were performed using Stata version 11.

## Results

**Figure 2:**
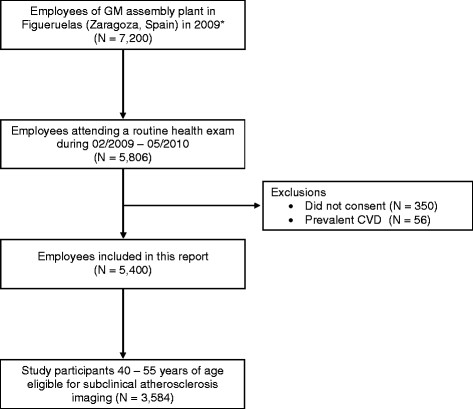
Flowchart of study participants, Aragon Workers Health Study.

Between February 2009 and May 2010, 5,775 workers attended the annual clinical exam, of whom 5,456 (94.5%) consented to participate in the study (Figure 2). After exclusion of 56 participants with prevalent CVD, the final sample size was 5,400. Most participants were involved in production jobs (manufacturing and assembly of automobile pieces and components) from four areas: presses, car body, painting, and final assembly. Other occupations included maintenance (plumbing, electricity, electronics, etc.), quality control (process laboratory and quality assurance), movement of materials, and administrative work. Most workers were involved in manual (blue collar) jobs (86.5% of men and 60.5% of women).

Among males, the average (SD) age, body mass index, and waist circumference were 49.3 (8.7) years, 27.7 (3.6) kg/m^2^ and 97.2 (9.9) cm, respectively (Table 1). The proportion of male participants who were overweight, obese, and current smokers were 55.0, 23.1, and 37.1%, respectively. The average systolic and diastolic blood pressure were 127.0 (14.7) and 83.8 (10.1) mmHg, with a prevalence of hypertension of 40.3%. The average levels of total cholesterol, HDL-cholesterol and triglycerides were 212.4 (37.6), 52.4 (11.0), and 148.8 (106.1) mg/dL, respectively, with a prevalence of hypercholesterolemia of 75.0%. The prevalence of diabetes was 7.4%.

**Table 1:** Aragon’ Workers Health Study (AWHS) baseline characteristics, 2009 – 2010

	**Men (N = 5,048)**		**Women (N = 351)**	
	**N**	**Mean (SD) or N (%)**	**N**	**Mean (SD) or N (%)**
Age (y)	5,048	49.3 (8.7)	351	40.8 (11.6)
Height (cm)	5,031	171.5 (6.5)	351	161.3 (6.9)
Weight (kg)	5,031	81.6 (11.6)	350	63.5 (10.2)
BMI (kg/m^2^)	5,014	27.7 (3.6)	350	24.4 (3.8)
Underweight		11 (0.2)		6 (1.7)
Normal		1,085 (21.6)		232 (66.3)
Overweight		2,759 (55.0)		83 (23.7)
Obese		1,159 (23.1)		29 (8.3)
Waist circ. (cm)	4,987	97.2 (9.9)	345	81.9 (9.9)
Systolic BP (mmHg)	4,892	127.0 (14.7)	337	111.4 (13.2)
Diastolic BP (mmHg)	4,892	83.8 (10.1)	337	76.4 (9.5)
Tot. cholesterol (mg/dL)*	5,048	212.4 (37.6)	351	204.3 (39.9)
HDL-cholesterol (mg/dL)*	5,048	52.4 (11.0)	351	66.5 (14.2)
Triglycerides (mg/dL) *	5,048	148.8 (106.1)	351	89.7 (75.5)
Glucose (mg/dL)*	5,048	98.3 (19.6)	351	91.2 (16.3)
Smoking habits	5,017		349	
Never		1,796 (35.8)		146 (41.8)
Former		1,359 (27.1)		46 (13.2)
Current		1,862 (37.1)		157 (45.0)
Hypertension	4,918	1,982 (40.3)	338	41 (12.1)
Hypercholesterolemia	5,048	3,788 (75.0)	351	209 (59.5)
Diabetes	5,048	371 (7.4)	351	2 (0.6)

Note: AWHS included 5,400 participants; 1 participant with missing sex data is not included in the Table.

* To convert to mmol/L multiply cholesterol levels by 0.0259, triglyceride levels by 0.0113, and glucose levels by 0.0555.

Among females, the average (SD) age, body mass index, and waist circumference were 40.8 (11.6) years, 24.4 (3.8) kg/m^2^ and 81.9 (9.9) cm, respectively (Table 1). The proportion of female participants who were overweight, obese, and current smokers were 23.7, 8.3, and 45.0%, respectively. The average systolic and diastolic blood pressure were 111.4 (13.2) and 76.4 (9.5) mmHg, with a prevalence of hypertension of 12.1%. The average levels of total cholesterol, HDL-cholesterol and triglycerides were 204.3 (39.9), 66.5 (14.2), and 89.7 (75.5) mg/dL, respectively, with a prevalence of hypercholesterolemia of 59.5%. The prevalence of diabetes was 0.6%.

The initial 587 study participants who completed all imaging exams (94.5% male) had an average (SD) age of 50.9 (3.6) years. The prevalence of carotid plaque, femoral plaque, coronary calcium score >1 to 100, and of coronary calcium score >100 was 30.3, 56.9, 27.0, and 8.8%, respectively. 67.7% of study participants had at least one plaque in the carotid or femoral arteries (Table 2).

**Table 2:** Prevalence of subclinical atherosclerosis in the initial 587 AWHS participants completing all imaging procedures

	**Age 40 – 50**	**Age 50 – 56**	**Overall**
Age, years	47.0 (2.5)	52.9 (2.5)	50.9 (3.6)
Carotid plaque	46 (18.5)	194 (35.7)	240 (30.3)
Femoral plaque	101 (39.6)	317 (56.0)	418 (50.9)
Coronary calcium			
Agatston score >1 to 100	48 (20.0)	159 (30.5)	207 (27.2)
Agatston score >100	8 (3.3)	59 (11.3)	67 (8.8)

Values in the Table are number (%), except for age [mean (SD)].

## Discussion

In AWHS, we recruited a cohort of working middle age men and women with a high prevalence of traditional risk factors and of subclinical atherosclerosis but with a low prevalence of overt CVD. At baseline, the AWHS population showed a high prevalence of practically all cardiovascular risk factors, and particularly of overweight and obesity. While there is no nationally representative study of the prevalence of cardiovascular risk factors in Spain, pooled analyses of local population-based studies have also identified a high prevalence of cardiovascular risk factors in the general population in Spain (Table 3) [5,6,8-10].

**Table 3:** Prevalence of traditional cardiovascular risk factors in large population studies in Spain

**Study**	**ERICE**[9]*	**DARIOS**[10]	**Sánchez-Chaparro et al**[31].
**Population**	Eight cross-sectional studies representative of the general adult population of the study’s target area	Studies representative of the general adult population of the study’s target area	Cross-sectional study health insurance company
**Period of data collection**	1992 – 2001	2000 – 2009	2004 – 2005
**Age range (years)**	45 – 64	35 – 74	16 – 74
**Sample size**			
**Men**	2,782	13,425	158,593
**Women**	3,412	15,462	58,321
**Current smoking (%)**			
**Men**	38.6	33.0	51.3
**Women**	6.5	21.0	43.8
**Hypertension (%)**			
**Men**	49.0	47.0	27.0
**Women**	50.3	39.0	8.8
Hypercholesterolemia (%)**			
**Men**	59.8	81.0	48.7
**Women**	59.6	79.0	40.6
**Diabetes (%)**			
**Men**	2.3	16.0	3.0
**Women**	1.1	11.0	0.6
**Obesity (%)**			
**Men**	15.9	29.0	18.3
**Women**	13.3	29.0	8.0

* The ERICE Study included participants >18 years old; data in the present table is restricted to participants 45 to 64 years old.

** Total cholesterol >200 mg/dL (5.2 mmol/L) in the ERICE Study and in the study by Sánchez-Chaparro et al., and >190 mg/dL (4.9 mmol/L) in the DARIOS Study.

The high prevalence of cardiovascular risk factors in the AWHS population, particularly among men, is also consistent with other studies of worker populations in Spain [31-36]. Among workers at a different car assembly plant in Spain [32], 69.3% of male workers had at least one cardiovascular risk factor. In a large sample of over 200,000 workers enrolled in a mutual insurance fund in 2004 – 2005 (average age 36.4 years) [31], the prevalence of smoking, overweight/obesity, hypertension, hypercholesterolemia, and diabetes were 51.3, 63.1, 27.0, 48.7, and 3.0%, respectively, in men, and 43.8, 29.2, 8.8, 40.6, and 0.6%, respectively, in women. Similarly, in the MESYAS Registry [34], the prevalence of workers who were current smokers or who had at least one metabolic abnormality were 51.4 and 65.5%, respectively. These high levels of cardiovascular risk factors occurred among relatively young populations under regular medical supervision, highlighting the need for non-medical, population-based approaches to cardiovascular risk control in these populations [37].

The high prevalence of cardiometabolic risk factors in the AWHS population is likely a consequence of the high prevalence of overweight/obesity. While there are no systematic health surveys with comparable measures of body mass index in Spain that allow for a detailed characterization of the obesity epidemic in the country [11], available data indicate that overweight/obesity has become a major health problem in Spain [11-13]. In the DARIOS Study, for instance, the average prevalence of overweight/obesity in population based studies conducted in Spain during 2000 – 2009 was 79% in men and 65% in women [10]. Survey data based on self-report suggest that the average body mass index of the Spanish population has increased continuously at least since the late 1980s and that the rate of growth has still not leveled off [12]. Indeed, the Spanish and Greek centers had the highest prevalence of obesity of all centers participating in the European Prospective Investigation into Cancer and Nutrition (EPIC) Study [38]. The high prevalence of overweight/obesity threatens the privileged situation of Spain in terms of CVD incidence [14]. Advancing the understanding of the link between obesity, cardiometabolic abnormalities and subclinical atherosclerosis is a major objective of the AWHS, well-suited to the high prevalence of excess adiposity in this population.

The AWHS uses a combination of non-invasive techniques, modeled after the HRP Bioimage study [19], to identify subclinical atherosclerosis. In the initial 587 cases, we found a higher prevalence of sublinical atherosclerotic plaques in the femoral arteries compared to the carotid arteries. In addition, the presence of carotid and femoral plaque was more common than the presence of coronary calcium. The use of ultrasound to identify atherosclerosis in the femoral arteries is significant innovation in AWHS compared to other cohorts. A comparison of AWHS to other landmark cohorts using non-invasive cardiovascular imaging [19,39-42] is available in Table 4. Coronary calcium scoring is the only novel cardiovascular biomarker that markedly adds discriminative power to risk scores based on traditional risk factors [15,43]. Carotid IMT is the most commonly used ultrasound parameter to assess subclinical atherosclerosis. Its usefulness has been limited by operator-dependency and high within subject variability [16], but the use of internal carotid artery maximum IMT or presence of plaque instead of average IMT may improve risk discrimination [44]. As in the HRP Bioimage Study, we will use a 3D ultrasound probe that may have a higher sensitivity for identifying carotid artery plaque compared to 2D IMT imaging. In the AWHS we will also perform abdominal and inguinal ultrasound scans to identify abdominal aortic aneurisms and iliac and femoral atherosclerotic plaques. Repeated ultrasound scans every 3 years and calcium coronary scans after 6 years will allow for a detailed characterization of the natural history of atherosclerosis in study participants and for the identification of novel determinants of its progression.

**Table 4:** Comparison of the Aragon Workers’ Health Study to landmark cohort studies of subclinical cardiovascular imaging

**Study**	**Aragon Workers’ Health Study (AWHS)**	**High-risk Plaque Initiative Biomage Study (HRP)**[19]	**Atherosclerosis Risk in Communities Study (ARIC)**[39,44]	**Cardiovascular Health Study (CHS)**[40]	**Multi-Ethnic Study of Atherosclerosis (MESA)**[41]
**Sample size**	5,400	7,687	15,792	5,888	5,800
**Study population**	Workers at an automobile assembly plant	Enrollees of a health plan free of CVD with at least 1 CVD risk factor	Probability sampling of household in participating communities	Probability sampling of Medicare elderly lists in participating communities	Volunteers free of CVD
**Age range (y)**	18 – 64	Men: 55 – 80	45 – 64	≥65	45 – 84
		Women: 60 – 80			
**Baseline period**	2009 – 2010	2008 – 2009	1987 – 1998	1989 – 1993	2000 – 2002
**Imaging methods at baseline**	US (2D + 3D) of carotid, abdominal aorta, femoral and iliac arteries	US (2D + 3D) of carotid arteries and abdominal aorta	US (2D) of carotid arteries	US (2D) of carotid arteries	US (2D) of carotid arteries and abodminal
			ABI	ABI	aorta
	CACS	CACS		Retinal photograph	CACS
	ABI	ABI			ABI
		CE-MRI			Cardiac MRI
		CT-angiography [18] F-FDG PET/CT			
**Follow-up visits**	Annual	No	Every 3 years	Annual through 1999	Every 2 years
				2005 – 2006	
**Imaging methods during follow-up**	Imaging methods repeated every 3 years	No	Imaging methods repeated every 3 years CE-MRI	Imaging methods repeated after 3 years	Imaging methods in subsamples
			Retinal photograph		CE-MRI

ABI: ankle-brachial blood pressure index; CACS: coronary artery calcium scoring; CE-MRI: contrast-enhanced magnetic resonance imaging; CT: computed tomography; CVD: cardiovascular disease; FDG: fluorodeoxyglucose; PET: positron emission tomography; US: ultrasound.

The AWHS provides an excellent opportunity to evaluate cardiometabolic factors and subclinical atherosclerosis in a middle-age Mediterranean population with high levels of overweight/obesity. Compared to other studies, the availability of annual follow-up visits of study participants will allow for a detailed evaluation of the trajectories of cardiometabolic parameters and subclinical atherosclerosis and their behavioral, environmental, and genetic determinants. The high response rate observed is a reflection of the long-standing collaboration between study investigators and personnel of the Medical Services of General Motors Spain. Consistent with previous collaborations of the research team, we anticipate a high follow-up rate. In addition, although the design of the AWHS has focused on cardiometabolic risk factors because of their high impact in this population, the cohort design of the AWHS will accommodate the simultaneous study of other health issues. Because of the annual study visits, the study is also well suited to incorporate ancillary studies or tests that allow for the evaluation of novel hypotheses.

As in any study, the findings of AWHS will be affected by a variety of limitations. The main threat of losses to follow-up corresponds to retiring workers and to workers who stop working due to health issues. Whenever workers terminate employment, we will register the reasons and will invite participants to follow-up visits at the AWHS clinic at the Hospital Miguel Servet in Zaragoza, Spain. In addition to selection biases, two factors will limit the generalizability of the study. First, the AWHS is based on a working population, and the findings may not apply to non-working groups. The inclusion of workers in the study, however, does not affect the attainment of the main objectives of the study as the disease processes evaluated in the study are not restricted to specific occupations. Second, the study population is composed largely, although not exclusively, of men, reflecting the sex distribution in the factory. As a consequence, AWHS will not be able to study cardiometabolic risk factors that affect differentially men and women.

In spite of its limitations, AWHS will provide novel information on the distribution and trajectories of risk factors and subclinical CVD in a population with a high prevalence of underlying risk factors but still with a low prevalence of clinical CVD. We expect to identify trajectories of risk factors that will provide hints for the control of the progression of early atherosclerosis and thus minimize the burden of disease in later years of life. Follow-up of this cohort will also allow the assessment of subclinical atherosclerosis progression and the link of disease progression to traditional and emergent risk factors.

## Competing interests

None of the authors had financial or non-financial competing interests related to this study.

## Authors’ contributions

JAC, EG, and VF designed the study. JAC, VA, MLeon, JLP and GS organized and supervised data collection and quality control issues related to clinical measures. MP, MLaclaustra, and JMO organized and supervised data collection and quality control issues related to laboratory measurements and biobanking. FC, JJB organized and supervised data collection and quality control issues related to imaging techniques. MLaclaustra and MLeon organized and supervised the study database. EG and MLaclaustra performed statistical analyses. JAC, EG BI, MLaclaustra, MLeon, JMO, MP, and GS integrated the study scientific committee. EG drafted the manuscript. All authors provided important intellectual revisions to the manuscript and read and approved the final manuscript.

## Pre-publication history

The pre-publication history for this paper can be accessed here:

http://www.biomedcentral.com/1471-2261/12/45/prepub
